# Income-Based Inequalities in Five-Year Survival after Coronary Artery Bypass Grafting and Percutaneous Coronary Intervention among Formally Employed Adults in Colombia: A Nationwide Cohort Study

**DOI:** 10.5334/gh.1494

**Published:** 2025-11-27

**Authors:** Andrés Felipe Patiño-Benavidez, Darío Echeverri, Carlos Eduardo Obando López, Nicolás Uribe Valencia, Giancarlo Buitrago

**Affiliations:** 1 Fundación Cardioinfantil—Instituto de Cardiología, Bogotá, Colombia; 2 Instituto de Investigaciones Clínicas, Facultad de Medicina, Universidad Nacional de Colombia, Bogotá, Colombia

**Keywords:** Administrative health data, Colombia, Coronary artery bypass grafting, Long-term survival, Percutaneous coronary intervention, Socioeconomic inequality

## Abstract

**Background:**

Myocardial revascularization by coronary artery bypass grafting (CABG) or percutaneous coronary intervention (PCI) lowers mortality, yet long-term outcomes may vary by socioeconomic status despite broadly similar access to care.

**Objective:**

To examine the association between income—measured in legal monthly minimum wages (MMW)—and five-year survival after revascularization in formally employed Colombians (2012–2018).

**Methods:**

This was a retrospective cohort study using linked national administrative datasets. Income was grouped into quartiles at cohort entry. The primary outcome was five-year mortality. Analyses were stratified by procedure (CABG, PCI). Multivariable Cox models were used to estimate adjusted hazard ratios (aHRs), controlling for age, sex, region, Charlson Comorbidity Index, recent acute myocardial infarction, valve surgery within 30 days, and pre-procedural cardiac rehabilitation (30 days). Socioeconomic gradients were summarized using the Relative Index of Inequality (RII) and the Slope Index of Inequality (SII; absolute difference per 100,000 patients).

**Results:**

Among 8,128 patients (mean age = 55.0 ± 9.3 years; 11.3% women), 2,131 underwent CABG and 5,997 underwent PCI. After CABG, five-year mortality was 13.2% in Q1 vs 7.8% in Q4 (p < 0.01); aHRs (vs Q1) were 0.60 (95% CI = 0.40–0.90) for Q2, 0.56 (0.38–0.84) for Q3, and 0.58 (0.38–0.88) for Q4 (all p ≤ 0.01). After PCI, mortality declined from 11.7% (Q1) to 6.5% (Q4) (p < 0.01); only Q4 remained significant after adjustment (aHR = 0.64; 95% CI = 0.49–0.82; p < 0.01). Inequality indices confirmed the gradient: for CABG, SII = 4.14 per 100,000 (95% CI = 3.30–4.98; p < 0.01) and RII = 1.97 (1.17–3.31; p = 0.01); for PCI, SII = 3.13 per 100,000 (2.74–3.52; p < 0.01) and RII = 1.81 (1.32–2.48; p < 0.01).

**Conclusions:**

Lower income is associated with worse five-year survival after myocardial revascularization, with larger absolute and relative inequalities after CABG than after PCI.

## Introduction

Myocardial revascularization is a cornerstone intervention for managing coronary artery disease (CAD), aimed at restoring myocardial perfusion, reducing cardiovascular morbidity, and improving survival (1,2). The two main revascularization strategies—coronary artery bypass grafting (CABG) and percutaneous coronary intervention (PCI)—are selected based on factors such as coronary anatomy, patient comorbidities, clinical presentation, and technical aspects (1,3,4,5,6). Despite advancements in revascularization techniques and postoperative care, socioeconomic disparities continue to influence both access to these procedures and long-term survival outcomes (7). Whether such disparities persist among formally employed populations in low- and middle-income countries (LMICs), where access is intended to be uniform, remains unclear.

In LMICs like Colombia, formally employed individuals generally have a socioeconomic advantage due to their stable income and legally mandated access to social security benefits, including healthcare, pensions, and occupational risk insurance (8). This group comprises public and private sector workers whose employment contracts meet legal criteria for formal labor (8). However, despite these benefits, socioeconomic status (SES) appears to be a critical determinant of health outcomes, particularly in specialized cardiovascular care (9).

SES has been consistently linked to disparities in cardiovascular care and survival following revascularization procedures (7,10,11,12,13,14,15,16,17,18,19,20,21,22,23,24,25,26,27,28,29,30,31). Evidence from the SWEDEHEART registry has demonstrated that patients with lower incomes and lower educational attainment are less likely to adhere to secondary prevention medications after CABG, leading to higher long-term mortality (32). A Swedish nationwide cohort study further confirmed that higher disposable household income is associated with improved survival after cardiac surgery, emphasizing the role of economic disparities in postoperative prognosis (33). Additionally, studies conducted in Canada and Québec have shown that patients from lower socioeconomic backgrounds experience reduced access to invasive cardiac procedures and worse post–myocardial infarction (MI) survival, even within publicly funded healthcare systems (10). Furthermore, research has directly examined the relationship between socioeconomic position, the likelihood of undergoing revascularization, and five-year survival after acute MI, consistently finding worse survival rates among individuals from lower socioeconomic strata (7).

Colombia’s healthcare system is based on mandatory health insurance, achieving coverage for approximately 98% of the population through two primary schemes. The contributory scheme covers formal workers and their families, accounting for about 46% of the population, while the subsidized scheme covers approximately 48% of the population, mainly comprising economically disadvantaged families. Additionally, special and exception schemes provide coverage for around 5% of the population, including military personnel, public school and university teachers, and other specific groups. Importantly, the benefit package provided by the contributory and subsidized schemes is identical, including myocardial revascularization procedures and the clinical management required for patient follow-up and complications. However, regional and economic disparities persist, particularly in access to specialized procedures like myocardial revascularization (34,35). Geographic factors play a crucial role, as urban regions such as Bogotá and the Central region benefit from better healthcare infrastructure, whereas rural and remote areas—including the Amazon and Pacific regions—face logistical challenges that limit timely access to specialized care (35). Given these contextual factors, it remains unclear whether formally employed individuals in Colombia experience socioeconomic disparities in survival following myocardial revascularization, despite their presumed uniform access to healthcare services.

This study examines the association between monthly minimum wages (MMW)—as a proxy for socioeconomic status—and five-year survival in formally employed individuals who underwent myocardial revascularization in Colombia between 2012 and 2018. By focusing on a population with presumed equitable healthcare access through the contributory system, this study evaluates whether income-related disparities continue to influence long-term survival outcomes. The findings contribute to a broader understanding of the social determinants of health, highlighting the persistent impact of socioeconomic inequalities on cardiovascular care and postoperative survival.

## Methods

### Study design and population

This retrospective cohort study included all formally employed individuals in Colombia who underwent myocardial revascularization between January 1, 2012, and June 30, 2018. Patients were identified using unique procedure codes for CABG and PCI, as listed in Supplementary File S1. Patients entered the cohort on the date of their first recorded revascularization and were followed for up to five years or until death, whichever occurred first. The data were accessed on January 15, 2025. The study protocol was reviewed and approved by the Ethics Committee of Fundación Cardioinfantil—Instituto de Cardiología (Acta No. 12, April 2, 2025), which waived the requirement for individual informed consent because only de-identified data were analyzed.

### Data sources

This study utilized three administrative databases, anonymized and linked by the Ministry of Health (MoH) using unique patient identifiers. The Base for the Study of Capitation Unit Sufficiency (UPC) provides comprehensive records of healthcare service utilization for individuals affiliated with the contributory scheme; it includes information on medical procedures, ICD-10 diagnostic codes, healthcare costs, service dates, geographic location, and healthcare providers. It covers approximately 20 million individuals, representing 88% of the scheme’s total population, and is recognized for its high degree of standardization and minimal underreporting (36). The Wages Database (PILA), managed by the MoH, contains payroll contribution records related to social security. This dataset provides accurate wage information for formally employed individuals; self-employed individuals report their income, whereas employers report wages for formal employees, which ensures higher data accuracy and reliability (37). These databases, widely utilized in epidemiological and economic research, were provided to the Clinical Research Institute of the Universidad Nacional de Colombia for research proposals. The National Death Registry (RUAF) compiles mortality information from official death certificates, including the date, location, and cause of death, along with sociodemographic variables such as sex, age, marital status, educational attainment, occupation, and ethnicity (38). International assessments have confirmed its reliability, with 91% of deaths formally recorded through death certificates as of 2016 (39).

### Study variables

This study examined socioeconomic disparities among patients undergoing myocardial revascularization. Sociodemographic and clinical variables were collected at cohort entry. The main exposure variable was socioeconomic status, defined by the formal income recorded at cohort entry month in the PILA database. Income was categorized into quartiles of legal MMW at the time of cohort entry. Income was measured at the time of the index revascularization to capture pre-procedure socioeconomic resources. Updating wages during follow-up risked conditioning on post-treatment changes (e.g., recovery-related work capacity), which could block mediation or introduce bias along the pathway from income, through care/adherence, to mortality. The primary outcome variable was five-year survival, defined as the time from cohort entry to either death or the end of the follow-up period, whichever occurred first. Mortality data were obtained from death certificates registered in the RUAF database. To control for potential confounders, we included sociodemographic and clinical variables measured at cohort entry. Sociodemographic variables included age, sex, health insurer, and geographic region (Colombia is divided into 32 administrative departments). Clinical variables were derived from the Charlson Comorbidity Index (CCI), an internationally validated tool adapted for use in Colombia by Oliveros et al. (40). The CCI was computed using ICD-10 diagnostic codes and included conditions such as acute myocardial infarction (AMI), congestive heart failure, peripheral vascular disease, stroke, dementia, chronic pulmonary disease, connective tissue disease, peptic ulcer disease, liver disease, diabetes, complications of diabetes, cancer, metastatic cancer, paraplegia, renal disease, severe liver disease, and HIV. To determine whether revascularization was performed due to AMI, we identified AMI diagnoses within the 30 days prior to the procedure. We also captured pre-procedural cardiac rehabilitation (CR), defined as ≥1 claim for supervised CR services recorded in the 30 days before the index revascularization. Additionally, any valve surgery performed within the 30 days following revascularization was analyzed to assess the severity of underlying cardiac disease. The revascularization type was defined based on procedural codes and categorized as either PCI or CABG.

### Statistical analysis

A descriptive analysis was conducted to summarize the sociodemographic and clinical characteristics of formally employed individuals undergoing myocardial revascularization. Continuous variables were expressed as means and standard deviations when normally distributed or as medians with interquartile ranges otherwise. Categorical variables were reported as absolute and relative frequencies. Analyses were stratified by wage quartiles, with income categories defined according to the dataset distribution to ensure balanced representation without relying on subjective cutoffs.

Differences in five-year mortality across wage quartiles were assessed by calculating mortality proportions and their corresponding 95% confidence intervals (CIs). Unadjusted and adjusted hazard ratios (HRs) for five-year survival were estimated using Cox models, fitted separately for PCI and CABG. The multivariable models adjusted for sex, age, year of revascularization, type of health insurer, geographic region, CCI, the presence of AMI within 30 days prior to the procedure, and the requirement for valve surgery. The proportional hazards assumption was verified using both formal statistical tests and graphical diagnostics.

Five-year survival curves were generated from the adjusted models, stratified by both wage quartiles and revascularization type, to visually demonstrate survival differences. To quantify the socioeconomic gradient in survival outcomes, we computed the Relative Index of Inequality (RII) and the Slope Index of Inequality (SII). The RII, derived via Poisson regression models, reflects the relative difference in mortality risk between the lowest and highest wage groups, whereas the SII represents the absolute difference (per 100,000 individuals) in predicted five-year mortality rates across these groups. Both indices were estimated using the four ordered wage quartiles and were adjusted for the same covariates included in the Cox models, following the structured regression framework proposed by Moreno-Betancur et al. (41).

All statistical analyses were performed using Stata v.18 MP under the Universidad Nacional de Colombia license. The study adhered to STROBE (Strengthening the Reporting of Observational Studies in Epidemiology) guidelines, ensuring transparent and thorough reporting (Supplementary File S2). Key variables, including the exposure (monthly wages), the outcome (all-cause mortality), and routinely collected covariates, were highly complete in the linked administrative sources. Analyses included the entire cohort with no exclusions due to missing data; given this completeness, no imputation was performed. Additionally, artificial intelligence–based language models, including OpenAI’s GPT-4o, were utilized to refine the manuscript’s clarity and style.

## Results

**Figure 1:**
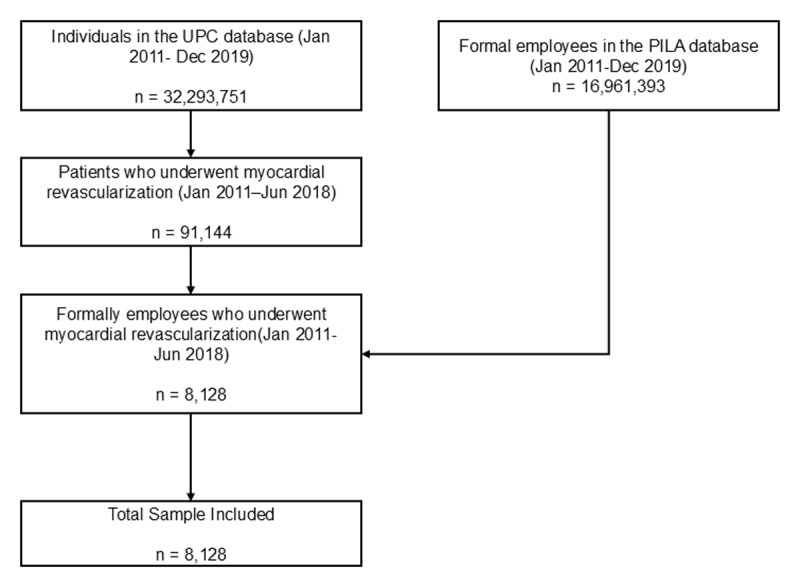
Flow diagram of cohort selection.

Between January 1, 2012 and June 30, 2018, 8,128 formally employed individuals underwent myocardial revascularization. The flowchart in Figure 1 provides additional details on the selection process. The overall mean age was 54.96 years (SD = 9.27). Patients in the lowest wage quartile (Q1) were slightly older (56.37 ± 10.01 years) than those in Q2 (54.37 ± 10.01 years), Q3 (54.16 ± 8.65 years), and Q4 (54.90 ± 8.10 years). Women represented 11.3% of the cohort, with the highest proportion in Q1 (13.9%) and the lowest in Q4 (8.2%; p < 0.01). Regarding age categories, 6.11% were <40 years, 18.76% were 41–50 years, 48.29% were 51–60 years, and 26.83% were >60 years. Q1 contained the greatest share of older adults (>60 years, 34.17%), whereas Q3 had the smallest (22.79%; p < 0.01). At 12 months after revascularization (same calendar month), 4.96% of patients moved to a lower-income quartile, approximately 80% remained in the same quartile, and approximately 15% could not be reassessed because of a change in health insurance scheme, which prevented wage identification in PILA for that month. A detailed overview of sociodemographic and clinical characteristics is provided in Table 1, and Supplementary File S3 includes a table with absolute standardized differences to evaluate differences in sociodemographic and clinical characteristics across income quartiles.

**Table 1:** Baseline characteristics of formally employed adults who underwent myocardial revascularization in Colombia (2012–2018), stratified by quartile of legal monthly minimum wages at cohort entry.


VARIABLE	TOTAL SAMPLE	Q1	Q2	Q3	Q4	p-VALUE

	N = 8128	N = 2040	N = 2020	N = 2010	N = 2058	

Age, mean (sd)	54.96 (9.27)	56.37 (10.01)	54.37 (10.01)	54.16 (8.65)	54.90 (8.10)	< 0.01

Sex, n (%)						< 0.01

Female	916 (11.27)	283 (13.87)	269 (13.32)	196 (9.75)	168 (8.16)	

Categorized Age, n (%)						< 0.01

Under 40 years old	497 (6.11)	91 (4.46)	168 (8.32)	135 (6.72)	103 (5.00)	

41 to 50 years old	1525 (18.76)	397 (19.46)	382 (18.91)	377 (18.76)	369 (17.93)	

51 to 60 years old	3925 (48.29)	855 (41.91)	950 (47.03)	1040 (51.74)	1080 (52.48)	

Over 60 years old	2181 (26.83)	697 (34.17)	520 (25.74)	458 (22.79)	506 (24.59)	

Region, n (%)						< 0.01

Atlántica	1152 (14.17)	283 (13.87)	253 (12.52)	290 (14.43)	326 (15.84)	

Bogotá DC	2976 (36.61)	617 (30.25)	740 (36.63)	804 (40.00)	815 (39.60)	

Central	2159 (26.56)	620 (30.39)	606 (30.00)	484 (24.08)	449 (21.82)	

Oriental	911 (11.21)	228 (11.18)	186 (9.21)	223 (11.09)	274 (13.31)	

Pacífica	908 (11.17)	287 (14.07)	234 (11.58)	204 (10.15)	183 (8.89)	

Orinoquía y Amazonía	22 (0.27)	5 (0.25)	1 (0.05)	5 (0.25)	11 (0.53)	

Charlson Index, n (%)						< 0.01

0	2187 (26.91)	441 (21.62)	584 (28.91)	559 (27.81)	603 (29.30)	

1–2	5049 (62.12)	1328 (65.10)	1239 (61.34)	1266 (62.99)	1216 (59.09)	

3–4	673 (8.28)	197 (9.66)	149 (7.38)	145 (7.21)	182 (8.84)	

≥5	219 (2.69)	74 (3.63)	48 (2.38)	40 (1.99)	57 (2.77)	

Revascularization (AMI), n (%)						< 0.01

Yes	4197 (51.64)	1138 (55.78)	1010 (50.00)	1051 (52.29)	998 (48.49)	

Valvular Surgery, n (%)						0.11

Yes	198 (2.44)	46 (2.25)	55 (2.72)	59 (2.94)	38 (1.85)	

Percutaneous Coronary Intervention, n (%)						0.49

Yes	5997 (73.78)	1479 (72.50)	1495 (74.01)	1491 (74.18)	1532 (74.44)	

Cardiac Rehabilitation						< 0.01

Yes	2197 (27.03)	550 (25.03)	479 (25.98)	573 (23.08)	595 (27.08)	

Insurer, n (%)						< 0.01

1	2068 (25.44)	493 (24.17)	523 (25.89)	551 (27.41)	501 (24.34)	

2	1058 (13.02)	328 (16.08)	292 (14.46)	254 (12.64)	184 (8.94)	

3	998 (12.28)	260 (12.75)	233 (11.53)	255 (12.69)	250 (12.15)	

4	691 (8.50)	172 (8.43)	190 (9.41)	154 (7.66)	175 (8.50)	

5	877 (10.79)	208 (10.20)	230 (11.39)	251 (12.49)	188 (9.14)	

6	545 (6.71)	84 (4.12)	101 (5.00)	108 (5.37)	252 (12.24)	

7	258 (3.17)	79 (3.87)	66 (3.27)	58 (2.89)	55 (2.67)	

8	702 (8.64)	192 (9.41)	166 (8.22)	165 (8.21)	179 (8.70)	

9	333 (4.10)	64 (3.14)	61 (3.02)	60 (2.99)	148 (7.19)	

10	225 (2.77)	56 (2.75)	74 (3.66)	74 (3.68)	21 (1.02)	

others	373 (4.59)	104 (5.10)	84 (4.16)	80 (3.98)	105 (5.10)	


Values are expressed as mean ± SD, median (IQR) or n (%), as appropriate. Percentages may not total 100 because of rounding. MMW = monthly minimum wage; AMI = acute myocardial infarction; CCI = Charlson Comorbidity Index; PCI = percutaneous coronary intervention; CABG = coronary-artery bypass grafting.

Geographically, 36.61% of patients resided in Bogotá, followed by the Central (26.56%) and Atlántica (14.17%) regions. In terms of comorbidity, 26.91% of the cohort had no Charlson comorbidities, with the highest share in Q4 (29.30%) and the lowest in Q1 (21.62%). A further 62.12% had a CCI of 1–2, most frequently in Q1 (65.10%) and least in Q4 (59.09%). Smaller proportions had more severe burden: 8.28% had an index of 3–4 (peaking at 9.66% in Q1 and falling to 7.21% in Q3) and only 2.69% had an index of 5 or greater (highest in Q1 at 3.63%, lowest in Q3 at 1.99%) (p < 0.01).

**Figure 2:**
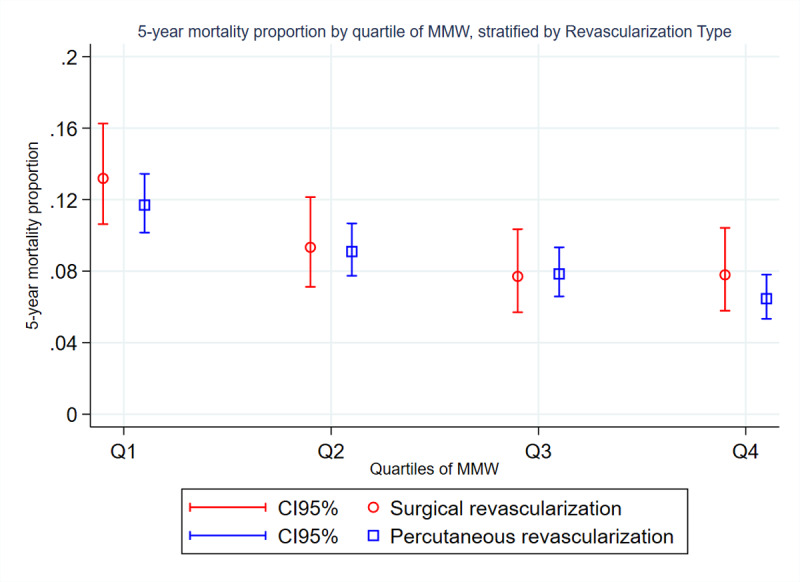
Five-year mortality proportion by quartile of monthly minimum wages, stratified by revascularization type.

During 5-year follow-up, an inverse socioeconomic gradient in mortality emerged. Among patients undergoing surgical revascularization, those in the lowest wage quartile (Q1) experienced a five-year mortality of 13.19% (95% CI = 10.63–16.25%), compared with 7.79% (95% CI = 5.79–10.42%) in the highest quartile (p < 0.01). In the PCI cohort, five-year mortality was 11.70% (95% CI = 10.16–13.44%) in Q1 vs 6.46% (95% CI = 5.34–7.81%) in Q4 (p < 0.001). Figure 2 illustrates these patterns, highlighting the consistent inverse relationship between wage level and survival across both revascularization methods.

**Figure 3:**
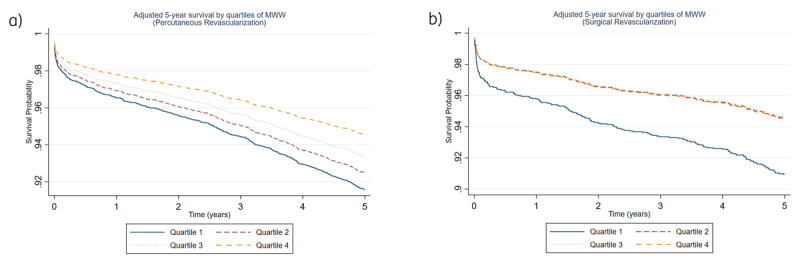
Adjusted five-year survival by quartiles of monthly minimum wages (MMW).

Table 2 shows that the association between wage quartiles and five-year survival differed by revascularization type. In the PCI cohort, unadjusted analyses revealed a graded decrease in mortality risk with higher wages: Q2 had an HR of 0.76 (95% CI = 0.61–0.96; p = 0.02), Q3 an HR of 0.65 (95% CI = 0.52–0.82; p < 0.01) and Q4 an HR of 0.53 (95% CI = 0.42–0.68; p < 0.01), all vs Q1. After multivariable adjustment, only Q4 remained statistically significant (adjusted HR = 0.64; 95% CI = 0.49–0.82; p < 0.01), whereas Q2 (0.90; 95% CI = 0.70–1.14; p = 0.39) and Q3 (0.79; 95% CI = 0.62–1.01; p = 0.06) did not. By contrast, in the CABG group the inverse gradient persisted after adjustment: compared with Q1, the adjusted HRs were 0.60 (95% CI = 0.40–0.90; p = 0.01) in Q2, 0.56 (95% CI = 0.38–0.84; p < 0.01) in Q3, and 0.58 (95% CI = 0.38–0.88; p = 0.01) in Q4. Figure 3a and b presents the adjusted survival curves for both revascularization methods.

**Table 2:** Five-year overall survival after percutaneous coronary intervention (PCI) and coronary artery bypass grafting (CABG) according to income quartile.


QUARTILE	5-YEAR OVERALL SURVIVAL OF PERCUTANEOUS REVASCULARIZATION						5-YEAR OVERALL SURVIVAL OF CABG					
	
	UNADJUSTED			ADJUSTED			UNADJUSTED			ADJUSTED		
			
	HR	CI95%	p	HR	CI95%	p	HR	CI95%	p	HR	CI95%	p

Q1	Ref	Ref	Ref	Ref	Ref	Ref	Ref	Ref	Ref	Ref	Ref	Ref

Q2	0.76	0.61–0.96	0.02	0.90	0.71–1.14	0.39	0.69	0.48–0.99	0.05	0.60	0.40–0.90	0.01

Q3	0.65	0.52–0.82	0.00	0.79	0.62–1.01	0.06	0.57	0.39–0.84	0.00	0.56	0.38–0.84	0.00

Q4	0.53	0.42–0.68	0.00	0.64	0.49–0.82	0.00	0.57	0.39–0.84	0.00	0.58	0.38–0.88	0.01


Unadjusted and adjusted hazard ratios (HR) were estimated with semi-parametric Cox proportional-hazards models. Adjustment variables: age (continuous), sex, year of procedure, health-insurance type, geographic region, Charlson Comorbidity Index, AMI ≤ 30 days before revascularisation, cardiac rehabilitation and valve surgery ≤ 30 days after revascularisation. 95% CI = confidence interval.

The RII and SII analyses confirmed a marked socioeconomic gradient in five-year survival (Table 3). For CABG, moving from the highest to the lowest wage group was associated with an SII of 4.14 per 100,000 (95% CI = 3.30–4.98; p < 0.01) and a 1.97-fold higher mortality at the bottom vs the top of the income distribution (RII = 1.97; 95% CI = 1.17–3.31; p = 0.01). For PCI, the corresponding difference was an SII of 3.13 per 100,000 (95% CI = 2.74–3.52; p < 0.01) with an RII of 1.81 (95% CI = 1.32–2.48; p < 0.01), indicating a smaller but statistically significant gradient; inequalities were more pronounced after CABG. These findings underscore a strong inverse relationship between wage level and survival, particularly after surgical revascularization.

**Table 3:** Relative and Slope Indices of Inequality (RII and SII) in 5-year mortality after myocardial revascularization.


INDEX	CABG			PCI		
	
	ESTIMATE	95% CI	P-VALUE	ESTIMATE	95% CI	P-VALUE

Relative Index of Inequality	1.97	1.17–3.31	0.01	1.81	1.32–2.48	0.00

Slope Index of Inequality (per 100,000)	4.14	3.30–4.98	0.00	3.13	2.74–3.52	0.00


RII represents the relative risk of death between the bottom and top of the income hierarchy; SII indicates the absolute percentage-point difference per 100 000 persons between those extremes. Both indices were derived from Poisson regression models adjusted for the same covariates used in the Cox models.

## Discussion

This study assessed the relationship between socioeconomic status, measured by MMW, and five-year survival among formally employed individuals undergoing myocardial revascularization in Colombia. Our findings highlight a significant socioeconomic gradient in survival outcomes, and although all patients were formally insured under Colombia’s contributory scheme, income-based differences in five-year survival persisted. The observed disparities persisted across both surgical (CABG) and percutaneous (PCI) revascularization modalities. Additionally, robust analyses using indices of relative and absolute inequality further confirmed these pronounced socioeconomic disparities, revealing substantially higher mortality risks among patients in the lowest income groups. To our knowledge, this is the first nationwide study conducted in an LMIC to document income-related inequalities in long-term survival following myocardial revascularization among formally employed adults, a population traditionally considered to have uniform access to healthcare services.

Our findings are consistent with previous studies conducted in high-income countries, where lower income and lower educational attainment have been linked to poorer adherence to secondary prevention therapies and higher mortality after revascularization procedures (7,10,11,12,13,14,15,16,17,18,19,20,21,22,23,24,25,26,27,29,30,31). The SWEDEHEART registry reported that patients with lower SES had reduced adherence to preventive cardiovascular medications, which was associated with higher mortality rates after CABG (32). Similarly, a Swedish nationwide cohort found an inverse association between disposable household income and survival after cardiac surgery, reinforcing the role of economic disparities in postoperative outcomes (33). Studies from Canada and Québec have also shown that even in publicly funded healthcare systems, patients from lower SES backgrounds experience reduced access to invasive cardiac procedures and worse survival after MI (10). Our study contributes to this growing body of evidence by showing that socioeconomic disparities in survival persist in a middle-income country, despite a contributory regime that has fewer barriers to accessing specialized care.

The observed disparities in survival may be influenced by several factors. First, although formal employment provides access to healthcare services, pensions, and occupational risk insurance, income disparities could still impact health-related behaviors, financial constraints for out-of-pocket expenses, and adherence to follow-up care. Second, regional differences in healthcare infrastructure may contribute to variations in post-revascularization outcomes, as urban centers such as Bogotá and the Central region have greater access to high-complexity cardiovascular services, while rural and remote areas—such as the Amazon and Pacific regions—face logistical barriers, physician shortages, and limited access to specialized care. Third, lower-income individuals may have higher baseline cardiovascular risk profiles, including greater burden of comorbidities (as reflected in the CCI), which could negatively influence long-term survival.

This study has several strengths. The reliability of the data is a crucial advantage, as it includes comprehensive information on income and formal employee status, with wage data mandatorily reported by employers to the PILA database under national regulations (37). In addition, the RUAF death registry provides extensive coverage—documenting over 90% of deaths nationwide—while the UPC database offers detailed records on healthcare utilization (36,39); both have been widely used in previous studies, reinforcing their validity for investigating healthcare inequalities (40,42,43,44,45).

Several limitations should be considered. Although administrative databases provide robust data on service utilization, they often lack detailed clinical and behavioral variables would refine risk adjustment and better capture disease severity, such as left ventricular function, angiographic severity and lesion complexity, perioperative risk scores, medication use and adherence, and lifestyle factors (smoking, alcohol use, diet), as well as hospital-level information (e.g., hospital experience/volume and quality metrics) and facility characteristics (urban vs. rural).

CR after revascularization plausibly lies on the causal pathway between income and survival (through access, ability to attend sessions, and adherence). We therefore included pre-procedure rehabilitation as a baseline covariate but did not adjust for post-procedure rehabilitation in the main models to avoid over-adjustment of a mediator. In addition, while hospital-level quality/volume indicators and urban–rural identifiers were unavailable, all multivariable models adjusted for geographic region at cohort entry, partially accounting for contextual differences across insurer networks and catchment areas. Because these contextual variables are not recorded as a function of individual wages and referral/coding practices in the contributory system are largely determined by insurer networks and geography rather than patients’ income, any misclassification of hospital or urban/rural characteristics is expected to be non-differential with respect to income and to bias the income–survival association toward the null, rendering our estimates conservative.

Moreover, this study focuses on a relatively privileged population in terms of health status and income, so the income-related disparities observed may be even more pronounced in other groups, such as informal employees or the unemployed, or in countries with higher overall inequality. Future studies should include these populations to evaluate how income affects survival among patients undergoing myocardial revascularization, as well as to explore the broader impact of income-related inequalities on cardiovascular disease outcomes among patients who do not undergo revascularization, and to investigate cause-specific mortality related to cardiovascular disease. Finally, despite adjusting for several confounding factors, the possibility of residual confounding from unmeasured or unknown variables cannot be completely excluded, highlighting the need for further research in populations facing more significant barriers to accessing healthcare. Future research should go beyond descriptive analyses and rigorously investigate the causal pathways by which income disparities lead to poorer clinical outcomes.

## Conclusions

This study provides evidence that income disparities significantly influence five-year survival after myocardial revascularization among formally employed individuals in Colombia. Although all patients were formally insured under Colombia’s contributory scheme, income-based differences in five-year survival persisted. Lower-income patients had higher mortality rates, suggesting that socioeconomic inequalities persist in cardiovascular care. These findings highlight the critical role of income-related disparities in long-term health outcomes, emphasizing the need for targeted policies to improve secondary prevention, optimize post-revascularization care, and address regional inequities in specialized cardiovascular services. Further research is needed to explore the impact of additional socioeconomic determinants on survival outcomes and to develop interventions aimed at reducing disparities in cardiovascular health in Colombia and other middle-income settings. These findings call for policy interventions that go beyond formal coverage, addressing structural barriers that perpetuate health inequalities—even within insured populations.

## AI Use

ChatGPT (GPT-4o) was employed solely for language editing; all content was verified and approved by the authors.

## Data Accessibility Statement

The administrative databases analyzed in this study (UPC, PILA, and RUAF) are held by the Colombian Ministry of Health and Social Protection. All records were fully anonymized by the Ministry before being released to the research team. The Ministry authorized the use of these data for scientific purposes; the official authorization letter is provided in Supplementary File S4. Interested investigators should e-mail a brief study protocol to the Ministry’s Technology of the Information and Communication Office (correo@minsalud.gov.co) and sign the standard data-use agreement issued by the Ministry.

## Additional File

The additional file for this article can be found as follows:

10.5334/gh.1494.s1 Supplementary Files. Supplementary File S1 to S4.

## Disclaimer

The views expressed in this publication are those of the author(s) and not necessarily those of the NIHR or the UK government.
