# Supplementary material for: Income-Based Inequalities in Five-Year Survival after Coronary Artery Bypass Grafting and Percutaneous Coronary Intervention among Formally Employed Adults in Colombia: A Nationwide Cohort Study
Source: Glob Heart. 2025 Nov 27;20(1):105. doi: 10.5334/gh.1494 (PMC12662161; doi:10.5334/gh.1494)
Supplement: Supplementary Files. — Supplementary File S1 to S4. [file gh-20-1-1494-s1.pdf]

Supplementary File S1 – Procedure-code list

Complete catalogue of Colombian national procedure codes (CABG and PCI) used to define the study cohort.

A. Surgical revascularisation — Coronary-artery bypass grafting (CABG)

361000	361100	361200	361300	361400	361501	361505	361601	361602	361603
361604	361605	361606	361607	361608	361609	361610	361611	361612	361613
361614	361615	361616	361617	361618	361619	361620	361621	361622	361623
361624	361701	361703	361801	361802	361803	361804	361805	361806	361807
361808	361809	361810	361811	361812	361813	361814	361815	361816	361817
361818	361819	361820	361821	361822	361823	361824	361901	361902	361903
361904	361905	361906	361907	361908	361909	361910	361911	361912	361913
361914	361915	361916	361917	361918	361919	361920	361921	361922	361923
361924	362100	362200	362300	363101	363200	363201	363202		

B. Percutaneous revascularisation — Percutaneous coronary intervention (PCI)

360101	360102	360104	360105	360106	360201	360202	360301	360401	360501
360601									

Notes:

1. Codes follow the Colombian CUPS (Clasificación Única de Procedimientos en Salud).

2. All CABG codes (section A) describe open-heart surgical bypass procedures, including combined valve/CABG interventions where the bypass component is explicit.

3. All PCI codes (section B) describe percutaneous transluminal coronary angioplasty with or without stent implantation.

4. The national CUPS catalogue provides official long-form descriptions; investigators may cross-reference these codes at https://www.minsalud.gov.co for full procedural detail.

Supplementary File S2 – STROBE checklist

Completed "Strengthening the Reporting of Observational Studies in Epidemiology" checklist for cohort studies.

	Item No	Recommendation	Page No
**Title and abstract**	1	(*a*) Indicate the study's design with a commonly used term in the title or the abstract	1-2
		(*b*) Provide in the abstract an informative and balanced summary of what was done and what was found	2
**Introduction**			
Background/rationale	2	Explain the scientific background and rationale for the investigation being reported	3-4
Objectives	3	State specific objectives, including any prespecified hypotheses	4
**Methods**			
Study design	4	Present key elements of study design early in the paper	4
Setting	5	Describe the setting, locations, and relevant dates, including periods of recruitment, exposure, follow-up, and data collection	4
Participants	6	(*a*) *Cohort study*—Give the eligibility criteria, and the sources and methods of selection of participants. Describe methods of follow-up<br>*Case-control study*—Give the eligibility criteria, and the sources and methods of case ascertainment and control selection. Give the rationale for the choice of cases and controls<br>*Cross-sectional study*—Give the eligibility criteria, and the sources and methods of selection of participants	4
		(*b*) *Cohort study*—For matched studies, give matching criteria and number of exposed and unexposed<br>*Case-control study*—For matched studies, give matching criteria and the number of controls per case	-
Variables	7	Clearly define all outcomes, exposures, predictors, potential confounders, and effect modifiers. Give diagnostic criteria, if applicable	5

Data sources/ measurement	8*	For each variable of interest, give sources of data and details of methods of assessment (measurement). Describe comparability of assessment methods if there is more than one group	*5-6*
Bias	9	Describe any efforts to address potential sources of bias	5-6
Study size	10	Explain how the study size was arrived at	-
Quantitative variables	11	Explain how quantitative variables were handled in the analyses. If applicable, describe which groupings were chosen and why	5
Statistical methods	12	(*a*) Describe all statistical methods, including those used to control for confounding	5-6
		(*b*) Describe any methods used to examine subgroups and interactions	-
		(*c*) Explain how missing data were addressed	-
		(*d*) *Cohort study*—If applicable, explain how loss to follow-up was addressed<br>*Case-control study*—If applicable, explain how matching of cases and controls was addressed<br>*Cross-sectional study*—If applicable, describe analytical methods taking account of sampling strategy	-
		(*e*) Describe any sensitivity analyses	-

Results

Participants	13*	(a) Report numbers of individuals at each stage of study—eg numbers potentially eligible, examined for eligibility, confirmed eligible, included in the study, completing follow-up, and analysed	6
		(b) Give reasons for non-participation at each stage	7
		(c) Consider use of a flow diagram	7
Descriptive data	14*	(a) Give characteristics of study participants (eg demographic, clinical, social) and information on exposures and potential confounders	7
		(b) Indicate number of participants with missing data for each variable of interest	-
		(c) *Cohort study*—Summarise follow-up time (eg, average and total amount)	5
Outcome data	15*	*Cohort study*—Report numbers of outcome events or summary measures over time	*7-8*
		Case-control study—Report numbers in each exposure category, or summary measures of exposure	-
		Cross-sectional study—Report numbers of outcome events or summary measures	-
Main results	16	(*a*) Give unadjusted estimates and, if applicable, confounder-adjusted estimates and their precision (eg, 95% confidence interval). Make clear which confounders were adjusted for and why they were included	7-8
		(*b*) Report category boundaries when continuous variables were categorized	-
		(*c*) If relevant, consider translating estimates of relative risk into absolute risk for a meaningful time period	-
Other analyses	17	Report other analyses done—eg analyses of subgroups and interactions, and sensitivity analyses	-
**Discussion**			
Key results	18	Summarise key results with reference to study objectives	8

Limitations	19	Discuss limitations of the study, taking into account sources of potential bias or imprecision. Discuss both direction and magnitude of any potential bias	9
Interpretation	20	Give a cautious overall interpretation of results considering objectives, limitations, multiplicity of analyses, results from similar studies, and other relevant evidence	9
Generalisability	21	Discuss the generalisability (external validity) of the study results	9
**Other information**			
Funding	22	Give the source of funding and the role of the funders for the present study and, if applicable, for the original study on which the present article is based	10

Supplementary File S3 Absolute standardized differences by quartile comparisons (reference Q1)

Variable	Absolute standardized differences (Q1 vs Q2)	Absolute standardized differences (Q1 vs Q3)	Absolute standardized differences (Q1 vs Q4)
**Age**	0.2	0.24	0.16
**Sex**			
Female	0.02	0.13	0.18
**Categorized Age**			
Under 40 years old	0.16	0.1	0.03
41 to 50 years old	0.01	0.02	0.04
51 to 60 years old	0.1	0.2	0.21
Over 60 years old	0.19	0.25	0.21
**Region**			
Caribbean	0.04	0.02	0.06
Bogotá D.C.	0.14	0.21	0.20
Central	0.01	0.14	0.20
Eastern	0.07	0	0.07
Pacific	0.07	0.12	0.16
Orinoquía & Amazonia	0.05	0	0.05
**Charlson Index**			
0	0.17	0.14	0.18
1–2	0.08	0.04	0.12
3–4	0.08	0.09	0.03
≥5	0.07	0.1	0.05
**Revascularization (AMI)**			
Yes	0.12	0.07	0.15
**Valvular Surgery**			
Yes	0.03	0.04	0.03
**Percutaneous Coronary Intervention**			
Yes	0.03	0.04	0.04

**Cardiac Rehabilitation**			
Yes	0.08	0.04	0.04
**Insurer**			
1	0.04	0.07	0.00
2	0.05	0.1	0.22
3	0.04	0	0.02
4	0.03	0.03	0.00
5	0.04	0.07	0.04
6	0.04	0.06	0.30
7	0.03	0.06	0.07
8	0.04	0.04	0.03
9	0.01	0.01	0.18
10	0.05	0.05	0.13
Others	0.05	0.05	0.00

Supplementary File S4 – Ministry approval letter
Official letter from the Colombian Ministry of Health and Social Protection authorising the research use of the anonymised UPC, PILA and RUAF databases.

La salud es de todos | Minsalud

[illegible]

Radicado No.: [illegible]
Fecha: **05-03-2019**
Página 1 de 2

Bogotá D.C.,

Doctor
GIANCARLO BUITRAGO
Profesor asociado Departamento de Cirugía
Instituto de Investigaciones Clínicas
Universidad Nacional de Colombia
gbuitragog@unal.edu.co
No registra
Bogotá D.C.

ASUNTO: Solicitud información salud, Rad.201842301411402

Cordial saludo.

En atención a su solicitud de información anonimizada para las fuentes de interés, nos permitimos informarle:

1. Para el conjunto de datos de: Afiliados a salud, PILA, Información de Servicios de Salud – Estudio de Suficiencia y Nacimientos/Defunciones – RUAF-ND del período 2010-2016, se autoriza al Departamento de Epidemiología Clínica y Bioestadística a realizar la entrega de este conjunto de datos (adjunto oficio de autorización, rad.201913000267381) al Instituto de Investigaciones Clínicas de la Universidad Nacional.

2. Para el conjunto de datos de MIPRES, es necesario que definan las variables de interés, para lo cual se sugiere revisar la Resolución 1885 de 2018.

3. El conjunto de datos de Afiliados a salud, PILA e Información de Servicios de Salud – Estudio de Suficiencia del año 2017 y RIPS 2011-2017 están siendo procesados y se realizarán entregas graduales, debido al tiempo de procesamiento que demandan y volumen que ocupan. En la medida que se

La salud es de todos | Minsalud

Al contestar por favor cite estos datos:
Radicado No.: **201913000349201**
Fecha: **21-03-2019**

Bogotá D.C.,

Doctor
GIANCARLO BUITRAGO
Profesor asociado Departamento de Cirugía
Instituto de Investigaciones Clínicas
Universidad Nacional de Colombia
gbuitragog@unal.edu.co
No registra
Bogotá D.C.

ASUNTO: Solicitud información salud, Rad.201842301411402

Cordial saludo.

Atendiendo a su solicitud de registros anonimizados relacionados con las fuentes de información de: Afiliados a Salud, nacimientos y defunciones e Información de Servicios de Salud – Estudio de Suficiencia de 2017 y dando alcance al oficio de respuesta con radicado 201913000267461, nos permitimos informarles que se encuentra disponible el conjunto de datos que se relacionan en la Tabla 1, los cuales fueron copiados en el dispositivo externo suministrado por ustedes.

Tabla 1 Archivos entregados (carpeta:MSyPS-SISPRO-SGD)

Nombre archivo	Número de registros	Descripción
Est_Suf_2017_I.zip	120.310.412	Servicios de salud y medicamentos reportados para el período enero-abril de 2017
Est_Suf_2017_I.zip	127.864.390	Servicios de salud y medicamentos reportados para el período mayo-agosto de 2017
Est_Suf_2017_I.zip	124.769.891	Servicios de salud y medicamentos reportados para el periodo septiembre-diciembre de 2017
ExtraccionAfilaiciones2017_2018.zip	104.982.027	Datos de las afiliaciones a salud con corte a junio de 2017 y 2018
ExtraccionDefuncionesND 2017_2018_2019	574.505	Defunciones registradas en RUAF-ND para el periodo 2017-01 a 2019-02. Se entrega el archivo con el PersonalD (Identificador único para el fallecido), MadreID (Identificador único para la madre del fallecido en muertes fetales [illegible]

La salud es de todos | Minsalud

Al contestar por favor cite estos datos:
Radicado No.: **201913000349201**
Fecha: **21-03-2019**

Nombre archivo	Número de registros	Descripción
		archivo con el PersonalD (Identificador único para el fallecido), MadreID (Identificador único para la madre del fallecido en muertes fetales y en menores de 1 año).

Fuente: Elaborado Grupo SGD - OTIC

La información está dispuesta en archivos comprimidos con una contraseña, se solicita enviar notificación de recibido desde el correo autorizado por ustedes al correo electrónico lrincon@minsalud.gov.co para enviar la clave correspondiente; el nombre del asunto deberá ser "Solicitud Clave radicado 201913000349201" de acuerdo a lo establecido en el procedimiento para este fin.

Por parte de esta Oficina se solicita socializar la existencia de estos datos al interior de la Universidad, en aras a facilitar su consulta por parte de los interesados y evitar reprocesos a nivel del Ministerio de esta misma información, optimizando de esta manera los recursos.

Se hace entrega de esta información en los términos de la Ley 1581 de 2012 – *por el cual se dictan disposiciones generales para la protección de datos personales* – y su uso debe garantizar el derecho de habeas data.

Finalmente, es importante resaltar que para el Ministerio de Salud y Protección Social y en particular para esta Oficina será de gran beneficio conocer los resultados de los proyectos de investigación que ustedes adelanten utilizando los datos entregados, por lo que estaremos atentos a conocer el resultado de esos proyectos.

Atentamente,

DOLLY ESPERANZA OVALLE CARRANZA
Jefe Oficina de Tecnología de la Información y la Comunicación

Elaboró: LuzR
Revisó/Aprobó: MariaE

C:\LRincon\Trabajo LRINCONM\MINSALUD 2012\ORFEO 2017\Usuarios Externos

La salud es de todos | Minsalud

Bogotá D.C.,

Doctor
GIANCARLO BUITRAGO
Profesor asociado Departamento de Cirugía
Instituto de Investigaciones Clínicas
Universidad Nacional de Colombia
gbuitragog@unal.edu.co
No registra
Bogotá D.C.

ASUNTO: Solicitud información salud, Rad.201842301411402

Cordial saludo.

Atendiendo a su solicitud de registros anonimizados relacionados con las fuentes de información de: Registro Individual de Prestación de Servicios de Salud - RIPS, nacimientos y defunciones y dando alcance al oficio de respuesta con radicado 201913000267461, nos permitimos informarles que se encuentra disponible el conjunto de datos que se relacionan en la Tabla 1, los cuales fueron copiados en el dispositivo externo suministrado por ustedes.

Tabla 1 Archivos entregados (carpeta:MSyPS-SISPRO-SGD)

Nombre archivo	Número de registros	Descripción
RIPS_UNAL_2011	187.799.363	Datos anonimizados de las prestaciones de servicios de salud – RIPS año 2011
RIPS_UNAL_2012	226.655.031	Datos anonimizados de las prestaciones de servicios de salud – RIPS año 2012
RIPS_UNAL_2013	206.980.922	Datos anonimizados de las prestaciones de servicios de salud – RIPS año 2013
RIPS_UNAL_2014	288.816.837	Datos anonimizados de las prestaciones de servicios de salud – RIPS año 2014

Nombre archivo	Número de registros	Descripción
RIPS_UNAL_2017•	280.398.850	Datos anonimizados de las prestaciones de servicios de salud – RIPS año 2017
RIPS_UNAL_2018	378.305.880	Datos anonimizados de las prestaciones de servicios de salud – RIPS año 2018
PILA_UNAL_2017	142.605.356	Datos anonimizados de cotizantes año 2017 según estructura de entregas anteriores
ExtraccionNacimientosND2011_2016	3.653.372	Nacimientos registrados en RUAF-ND para el periodo 2011-01 a 2016-12. Se entrega el archivo con el PersonalD (Identificador único para la madre del nacido)
ExtraccionDefuncionesND2011_2016	1.275.657	Defunciones registradas en RUAF-ND para el periodo 2011-01 a 2016-12. Se entrega el archivo con el PersonalD (Identificador único para el fallecido), MadreID (Identificador único para la madre del fallecido en muertes fetales y en menores de 1 año).

Fuente: Elaborado Grupo SGD - OTIC

La información está dispuesta en archivos comprimidos con una contraseña en el dispositivo dispuesto por ustedes. Se solicita enviar notificación de recibido desde el correo autorizado por ustedes al correo electrónico lrincon@minsalud.gov.co para enviar la clave correspondiente; el nombre del asunto deberá ser "Solicitud Clave radicado 201913000640321" de acuerdo a lo establecido en el procedimiento para este fin.

Por parte de esta Oficina se reitera en la solicitud de socializar la existencia de estos datos al interior de la Universidad, en aras a facilitar su consulta por parte de los interesados y evitar reprocesos a nivel del Ministerio de esta misma información, optimizando de esta manera los recursos.

Se hace entrega de esta información en los términos de la Ley 1581 de 2012 – *por el cual se dictan disposiciones generales para la protección de datos personales* – y su uso debe garantizar el derecho de habeas data.

La salud es de todos | Minsalud

Finalmente, tal y como se ha manifestado en otras oportunidades, para el Ministerio de Salud y Protección Social y en particular para esta Oficina será de gran beneficio conocer los resultados de los proyectos de investigación que ustedes adelanten utilizando los datos entregados, por lo que estaremos atentos a conocer el resultado de esos proyectos.

Atentamente,

DOLLY ESPERANZA OVALLE CARRANZA
Jefe Oficina de Tecnología de la Información y la Comunicación

Elaboró: LuzR
Revisó/Aprobó: MariaE

C:\LRincon\Trabajo LRINCONM\MINSALUD 2012\ORFEO 2018\Usuarios Externos \UNal
